# Tamoxifen triggers the *in vitro* release of neutrophil extracellular traps in healthy horses

**DOI:** 10.3389/fvets.2022.1025249

**Published:** 2023-01-06

**Authors:** Constanza Salinas, Kassandra Barriga, Alejandro Albornoz, Pablo Alarcon, John Quiroga, Benjamín Uberti, José Sarmiento, Claudio Henriquez, Pamela Ehrenfeld, Rafael A. Burgos, Gabriel Moran

**Affiliations:** ^1^Instituto de Farmacología y Morfofisiología, Facultad de Ciencias Veterinarias, Universidad Austral de Chile, Valdivia, Chile; ^2^Instituto de Ciencias Clínicas Veterinarias, Facultad de Ciencias Veterinarias, Universidad Austral de Chile, Valdivia, Chile; ^3^Instituto de Fisiología, Facultad de Medicina, Universidad Austral de Chile, Valdivia, Chile; ^4^Instituto de Anatomía, Histología y Patología, Facultad de Medicina, Universidad Austral de Chile, Valdivia, Chile

**Keywords:** Neutrophil extracellular traps, tamoxifen, horses, *sprNETs*, *aggNETs*

## Abstract

Neutrophils display an array of biological functions including the formation of neutrophil extracellular traps (NETs), web-like structures specialized in trapping, neutralizing, killing and preventing microbial dissemination within the host. However, NETs contribute to a number of inflammatory pathologies, including severe equine asthma. Tamoxifen (TX) is a selective estrogen receptor modulator which belongs to the triphenylethyllenes group of molecules, and which is used as a treatment in all stages of estrogen-positive human breast cancer. Our previous results suggest that tamoxifen can modulate neutrophil functionality and promote resolution of inflammation; this would partly explain the clinical beneficial effect of this drug in horses with airway inflammation. Enhanced NETs production has been reported with tamoxifen use in humans, but minimal data exists regarding the drug's effect on NETs in horses. The aim of this study is to assess the *in vitro* effect of TX on NETs formation from peripheral blood of healthy horses. Five clinically healthy mixed-breed adult horses were enrolled in the study. For this, cellular free DNA quantification, immunofluorescence for the visualization of NETs, assessment of different types of NETs, and detection of mitochondrial superoxide. TX induced NETs formation at a concentration of 10 uM. Our results show that only two types of NETs were induced by TX: 95% spread NETs (*sprNETs*) and 5% aggregated NETs (*aggNETs*). Furthermore, induction of these NETs could be influenced by mitochondrial ROS. Future research should involve an *In vivo* study of horses with severe asthma and TX treatment, to evaluate BALF neutrophil NET formation. In conclusion, this *in vitro* study suggests that the resolution of inflammation by TX in horses with airway inflammation is due to inhibition of other neutrophilic functions but not to NET formation.

## 1. Introduction

Neutrophils, the most abundant and important cell component of the innate immune system play a major role in host defense as the first cellular type to be recruited to fight infection and inflammation (1, 2). They are the most abundant leukocytes in circulation and are first recruited to the infected sites. Here, neutrophils destroy pathogens *via* generation of reactive oxygen species (ROS), phagocytosis, and formation of neutrophil extracellular traps (NETs) (3). NETs are large, web-like structures composed of decondensed chromatin associated with histones and decorated with multiple antimicrobial proteins including elastase (NE) and myeloperoxidase (MPO) (4). These extracellular structures trap, neutralize, kill and prevent microbial dissemination within the host (5). The regulated process of NETs formation (NETosis) depends on the cellular mechanism triggered and activated by a wide range of stimuli (6).

Despite their protective role, dysregulated or excessive NETs formation can be detrimental to surrounding tissues and contribute to many immune-related diseases (5, 6). NETs can play a pathogenic role in many infectious and non-infectious human diseases, including respiratory cases with massive influx of neutrophils into the airways (7, 8). Excessive NETs release is particularly deleterious in lung diseases because NETs can easily expand in the alveolar space, causing lung injury (8), and epithelial and endothelial cell death (9). In horses, inflammatory processes are characterized by a strong neutrophilic response (2), as observed in most asthma-affected horses (10). The presence of NETs in the bronchoalveolar lavage fluid (BALF) from asthma cases but not in the fluid from healthy or remission horses (11), suggests that exacerbated NETosis may play a role as a possible and important promotor of the disease (12).

Tamoxifen (TX), a selective estrogen receptor modulator which belongs to the triphenylethyllenes group of molecules, is used as a treatment in all stages of estrogen-positive human breast cancer. It has potent anti-inflammatory effects: we have described an early pro-apoptotic effect of tamoxifen in granulocytic cells, both *in vitro* with peripheral blood and BALF (13), and *in vivo* (14); in addition, we showed that tamoxifen and its metabolites can activate the intrinsic apoptotic pathway in equine neutrophils (15). Our laboratory group has also shown that tamoxifen has an inhibitory action on respiratory burst, chemotaxis and chemokinesis of equine neutrophils in a dose-dependent manner (16, 17), and that tamoxifen induces efferocytosis by macrophages and modulates immune function through an estrogen-independent mechanism (18, 19). *In vivo*, our group demonstrated that horses with airway inflammation treated with 0.25 mg/kg PO of tamoxifen for five consecutive days display a significant decrease in BALF neutrophils and concomitant improvement in their clinical status in a manner similar to, or even better than that observed in hoses treated with dexamethasone (14). An independent group also found that tamoxifen produces an improvement in airway resistance in horses with exacerbated severe equine asthma, although no significant improvement in BALF neutrophil counts was observed in that study (20). Pharmacokinetic studies conducted by our laboratory showed that it is possible to measure tamoxifen as well as its main metabolites in blood as described in other species (21).

Since their first description in 2004, NETs are now known to play a role in physiology and pathology of different diseases (22), but the detailed mechanisms underlying NETs formation are not fully elucidated in horse neutrophils. Moreover, the effect of TX on NETs production in horses remains unknown. Therefore, the aim of this study is to assess the *in vitro* effect of tamoxifen on NETs formation from peripheral blood of healthy horses.

## 2. Materials and methods

### 2.1. Animals

Five clinically healthy mixed-breed adult horses (four mares and one gelding, body weight 420–450 kg, age 8–12 years) belonging to and housed at the Universidad Austral de Chile veterinary teaching hospital were enrolled in this study. All animals were housed on pasture and fed grass with free access to water. Physical examinations were performed before sample collection by qualified veterinarians to ensure animals' health. Each horse was sampled once, and each sample was analyzed independently. All procedures were approved by the Universidad Austral de Chile Bioethics Committee for the Use of Animals in Biomedical Research (approval resolution n° 348/2019).

### 2.2. Neutrophil isolation

The isolation of blood leukocytes was performed as previously described by our group (14, 16). Briefly, 10 mL of blood obtained by jugular venipuncture was placed in sterile tubes containing 1 mL of 3.8% w/v trisodium citrate. Blood was placed on a discontinuous density gradient (Percoll^®^, GE Healthcare), with 4 mL of 85% Percoll in the bottom of a 15 mL tube and 4 mL of 70% Percoll above. After centrifugation (45 min, 670 × g), the upper layer containing mononuclear cells, and the lower layer containing granulocytes, were aspirated for further processing. Granulocyte cell purity and *via*bility were assessed by flow cytometry (BD FACS Canto II). Viability was assessed using an Annexin V assay as previously described by our group (14). Cells were subsequently prepared for bioassays.

### 2.3. Cellular free DNA quantification

Quantification of extracellular free DNA was measured by spectrofluorometry. PMNs (1 × 10^6^) resuspended in Hanks' Balanced Salt Solution (HBSS, 136 mM NaCl, 5.3 mM KCl, 5.5 mM glucose, 1 mM MgCl_2_, 10 mM HEPES, 1.2 mM CaCl_2_, pH 7.4) were incubated in the presence of tamoxifen (TX, 10 μM, Sigma-Aldrich) or DMSO (0.1%, drug vehicle) for 30 min at 37°C with 5% CO_2_. Then, to stimulate NETs formation (positive control), cells were incubated with phorbol-12-myristate-13-acetate (PMA; 200 nM, Merck Millipore) for 1 h at 37°C with 5% CO_2_. Next, micrococcal nuclease (5U/well, New England Biolabs, Ipswich, MA, USA) was added to each sample, and incubated for 15 min at 37°C with 5% CO_2_. Cells were centrifugated at 300 × g for 5 min, and 100 μL of the supernatants were collected and transferred to a 96-well plate. PicoGreen^TM^ (1:200, Invitrogen, Carlsbad, CA, USA) was added to each well, followed by a 5 min incubation at RT. DNA content and NETs formation was analyzed using an automated Varioskan Flash Reader (Thermo Fisher Scientific, MA, USA) at 485 nm excitation and 530 nm emission.

### 2.4. NETs induction

Fresh isolated blood-derived equine polymorphonuclear cells were resuspended in RPMI medium (Cytiva,HyClone) supplemented with 2% fetal bovine serum. 2.5 × 10^5^ cells/wells (24 wells plate) were then seeded on 12 mm glass coverslips (pre-treated with 0.03% polylysine) and treated with tamoxifen (TX 10 μM; Sigma-Aldrich) or phorbol-12-myristate-13-acetate (PMA 200 nM; Merck Millipore) for 120 min at 37 °C with 5% CO_2._ Culture slides were fixed in 2% paraformaldehyde and permeabilized with 0.1% Triton X-100, in PBS.

### 2.5. Immunofluorescence staining for NETs

NETs from samples on point 2.4 were evaluated using immunofluorescence. Fixed cells were blocked with 3% bovine serum albumin (#9048-46-8, Sigma Aldrich) for 30 min. After this time, coverslips were incubated overnight at 4°C in the presence of rabbit anti-Histone H4 (Citrulline 3) (07-596, 1:200, Sigma-Aldrich). Next day, coverslips were washed three times with 1X PBS-T (0.05% Triton X-100) and then incubated for 2 hours at room temperature with anti-rabbit IgG (H+L) conjugated to Alexa Fluor 594^®^ (A-21070, 1:1000, Invitrogen). After this time and three washes with 1X PBS-T, nuclei acids were stained with PicoGreen™ (1:1000, Invitrogen) for 30 min at room temperature. Finally, coverslips were washed with 1X PBS-T and mounted using fluorescence mounting medium (Dako North America, Inc). Epifluorescence observations and photo documentation were performed using a Leica DM 2000 led microscope (Leica, Germany) equipped with a digital camera and ocular software (QImagin, Canada). Images were captured with settings kept consistent during collection of all images and processed with ImageJ software. Quantification of NETs was assessed with the DNA area and NETosis analysis (DANA) as described by Rebernick et al. (23). Data is expressed as NETosis percentage (%) and DNA area (μm^2^).

### 2.6. Assessment of different NETs types

Four randomly selected images from each experimental condition were then microscopically analyzed according to the typical morphological features according to Muñoz-Caro et al. (24) and Lange et al. (25): diffuse NETs (*diffNETs*), aggregated NETs (*aggNETs*) and spread NETs (*sprNETs*). The *diffNETs* are described as consisting of a globular and compact form with a size of 25–28 nm diameter. Whereas, *sprNETs* were observed consisting of smooth and elongated web- like structures composed exclusively by thin fibers with a diameter of 15–17 nm. The *aggNETs*, being defined as clusters of NET-like structures with a “ball of yarn” morphology and a size of more than 20 μm in diameter. Within each sample, structures with the specific features described above were counted.

### 2.7. Detection of mitochondrial superoxide

For measurement of mitochondrial ROS (mtROS) release, 1 × 10^6^ neutrophils were incubated with 10 μM rotenone (Tocris Bioscience, Bristol, UK), 200 μm MitoTEMPO (Cayman Chemical, Ann Arbor, MI, USA), 10 μM TX or TX plus rotenone and loaded with 2.5 μM MitoSOX™ Red Mitochondrial Superoxide Indicator (Invitrogen™, Thermo Fisher Scientific, Waltham, MA, USA) at 37 °C for 30 min. After incubation, MitoSOX™ fluorescence intensity was measured using a BD FACSCanto II flow cytometer (Becton Dickinson, Franklin Lakes, NJ, USA) at wavelengths of 510 nm excitation and 580 nm emission.

### 2.8. Statistical analysis

Results are presented as mean ± standard deviation (SD). Kolmogorov–Smirnov tests showed that the data were normally distributed. A one-way ANOVA was performed between treatments for cellular free DNA quantification, followed by a Tukey's HSD *post hoc* when significant differences were found. For DNA area and NETosis analysis (DANA), DNA area (μm^2^) was analyzed with a one-way ANOVA test followed by a Tukey's HSD *post hoc* when significant differences were found. Mitochondrial superoxide was analyzed with a one-way ANOVA test followed by a Tukey's test. Statistical analyses were performed with the commercial software GraphPad Prism for windows (GraphPad PRISM software v9.1.0, La Jolla California, USA). Statistical significance was defined at a *p*-value < 0.05.

## 3. Results

### 3.1. Equine neutrophils release cf-DNA in response to TX

**Figure 1:**
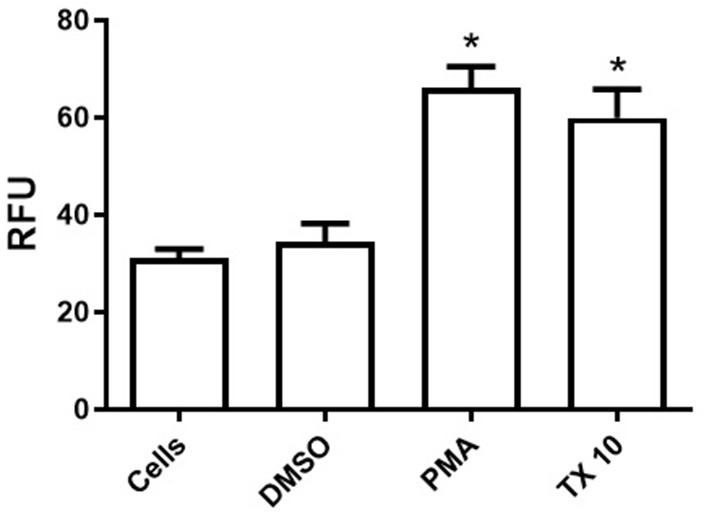
Tamoxifen (TX) triggers the release of cellular free-DNA (cf-DNA) from peripheral blood neutrophils of adult healthy horses. NETosis was quantified by cellular free-DNA measured in the supernatants of fresh-blood isolated neutrophils in the presence or absence of stimuli by spectrofluorometry. Unstimulated control cells (HBSS) and cells treated with DMSO (0.1%), PMA (200 nM) and TX (10 μM) were evaluated. Data is presented as means ± standard deviation (SD). RFU, relative fluorescence units;^*^p < 0.05; *n* = 5.

Our results shows that tamoxifen (TX) acts as a NET-inducing factor in equine polymorphonuclear cells of healthy adult horses. *In vitro* production of cf-DNA, quantified in the supernatants of PMN samples by spectrofluorometry, differed significantly between unstimulated cells and those treated with PMA (*p* < 0.05) or TX (*p* < 0.05) (Figure 1). The same effect was observed between the drug vehicle treated cells (DMSO) when compared to both, PMA (*p* < 0.05) and TX (*p* < 0.05) treated cells (Figure 1). Both stimuli showed a similar and significant increase in extracellular free-DNA concentrations, with no significative differences between PMA and TX treated cells (*p* > 0.05) (Figure 1). No statistically significant differences were observed between control cells and those incubated with DMSO (*p* > 0.05) (Figure 1).

### 3.2. TX induces NETs-type structures in equine neutrophils

**Figure 2:**
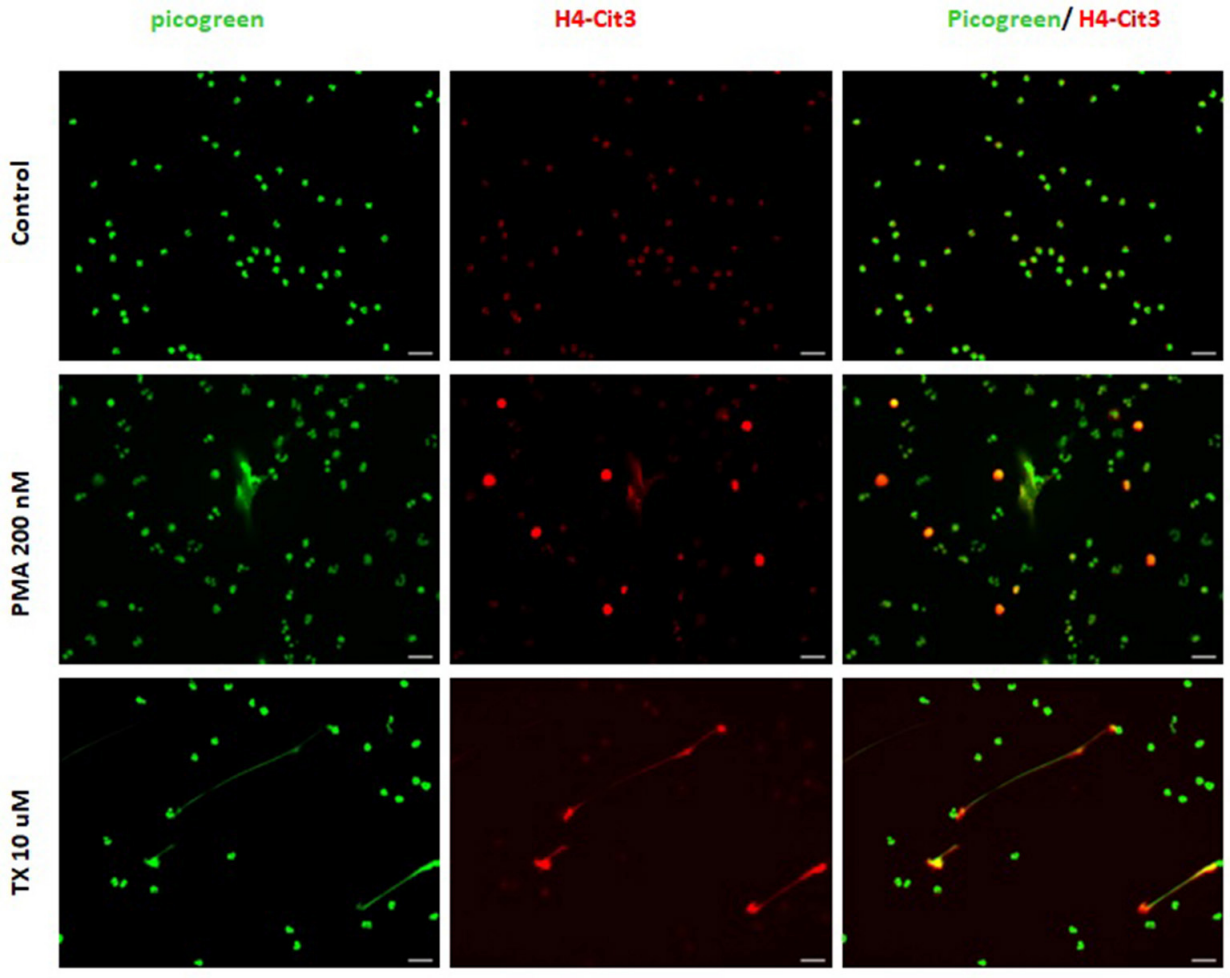
Tamoxifen (TX) stimulates equine PMNs NETosis. Representative fluorescence of equine PMNs treated with TX (10 μM) or PMA (200 nM) and marked with DNA staining PicoGreen^TM^. Strand-like NETs structures marked with PicoGreen^TM^ (green) and citrullinated histone H4 antibody (red) for control and treated cells with TX (10 μM) and PMA (200 nM). Scale bars = 30μm.

**Figure 3:**
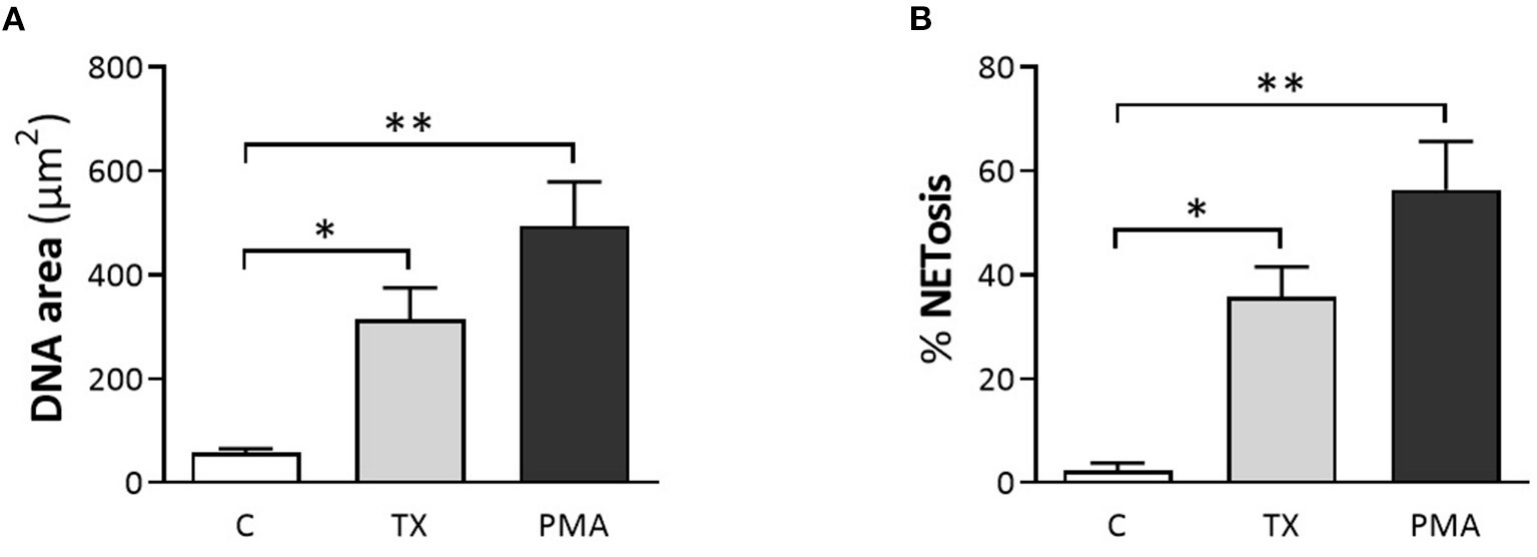
Tamoxifen (TX) induced the formation and release of NETs quantified by DNA area and NETosis (DANA) analysis. **(A)** Quantification of DNA area (μm^2^) and **(B)** percentage of NETosis for the three conditions studied. PMA (200 nM) was used as a positive control. Data are presented as mean ± standard deviation (SD). ^*^p < 0.05, ^**^p < 0.01; *n* = 5.

Consistent with these findings, immunofluorescence samples marked with the DNA staining PicoGreen™, showed positive DNA extrusion consistent with NETs structures for PMNs samples treated with TX or PMA. Immunofluorescence samples incubated with citrullinated histone H4 antibody showed positive DNA (PicoGreen™) and citrullinated H4 antibody staining for both TX and PMA treated cells. For control cells, minimal DNA strands-like structures and minimal positive marks for citrullinated histone H4 antibody were observed (Figure 2). For the DANA analysis, our results showed a statistically significant increase in the quantification of DNA area (μm^2^) for treated cells with TX (*p* < 0.05) and PMA (*p* < 0.01) when compared to unstimulated control cells (Figure 3). The same tendency was observed for NETosis percentage quantification, where a significant increase was observed for both TX (*p* < 0.05)and PMA (*P* < 0.01) treated cells when compared to the control cells (Figure 3).

### 3.3. Tamoxifen induces different types of NETs

**Figure 4:**
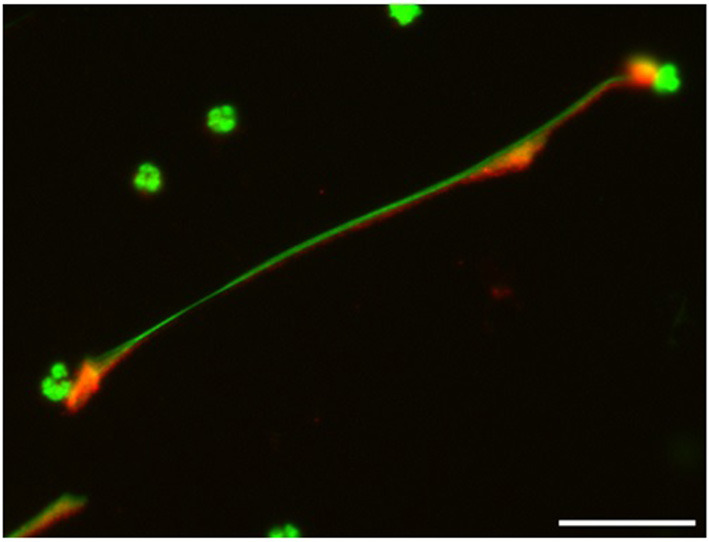
Tamoxifen triggers netosis mainly of the *sprNETS* type. Representative fluorescence of equine PMNs treated with TX (10 μM) and marked with DNA staining PicoGreen^TM^. Strand-like NETs structures marked with PicoGreen^TM^ (green) and citrullinated histone H4 antibody (red). Note the formation of *sprNETs* (consist of smooth, elongated, cobweb-like structures of decondensed chromatin, composed exclusively of fine fibers). Scale bars = 30μm.

**Figure 5:**
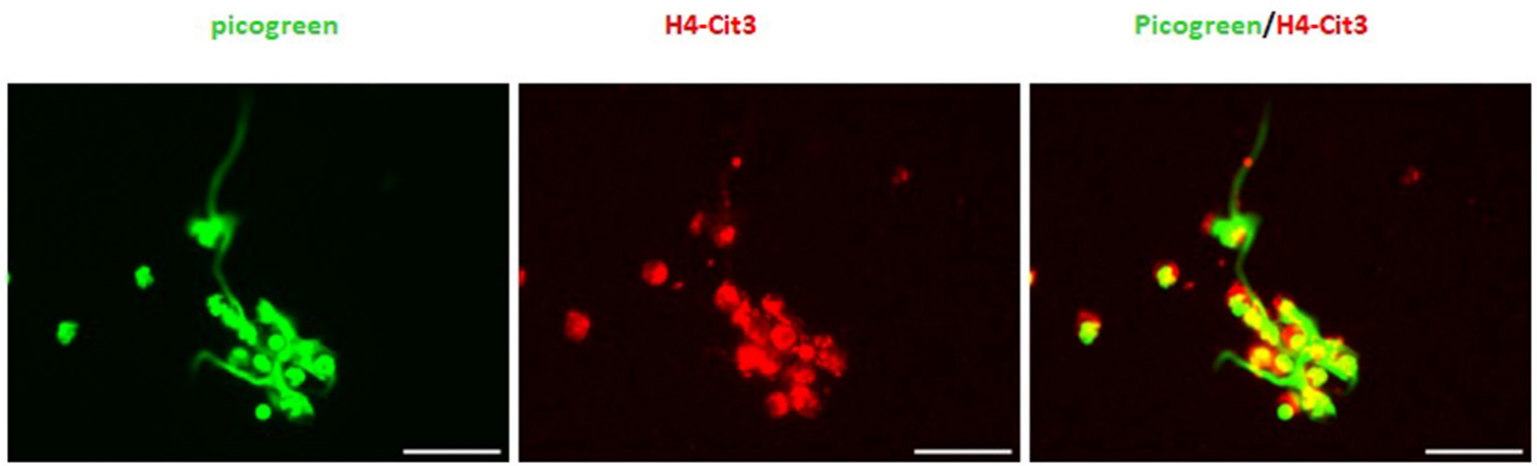
Tamoxifen triggers *aggNETs* type netosis in a very low percentage. Representative fluorescence of equine PMNs treated with TX (10 μM) and marked with DNA staining PicoGreen^TM^. Strand-like NETs structures marked with PicoGreen^TM^ (green) and citrullinated histone H4 antibody (red). Note the formation of *aggNETs* defined as clusters of NET-like structures with a “ball of yarn” morphology.

In general, NETs can manifest in different morphological forms, i.e., as *diffNETs, aggNETs* and *sprNETs*. Our results show that only two types of NETs were induced by TX. Morphological analysis showed that TX induced mainly *sprNETs* and to a lesser extent *aggNETs*. The *sprNETs* consist of smooth, elongated, cobweb-like structures of decondensed chromatin and antimicrobial proteins and are composed exclusively of fine fibers (Figure 4). The *aggNETs*, which are defined as clusters of NET-like structures with a “ball of yarn” morphology (Figure 5) (26). Quantification of the total samples showed that TX-induced *sprNETs* were 95%, while TX-induced *aggNETs* were only 5% of the total. No other NETs were found in the samples.

### 3.4. TX induces mitochondrial ROS release

**Figure 6:**
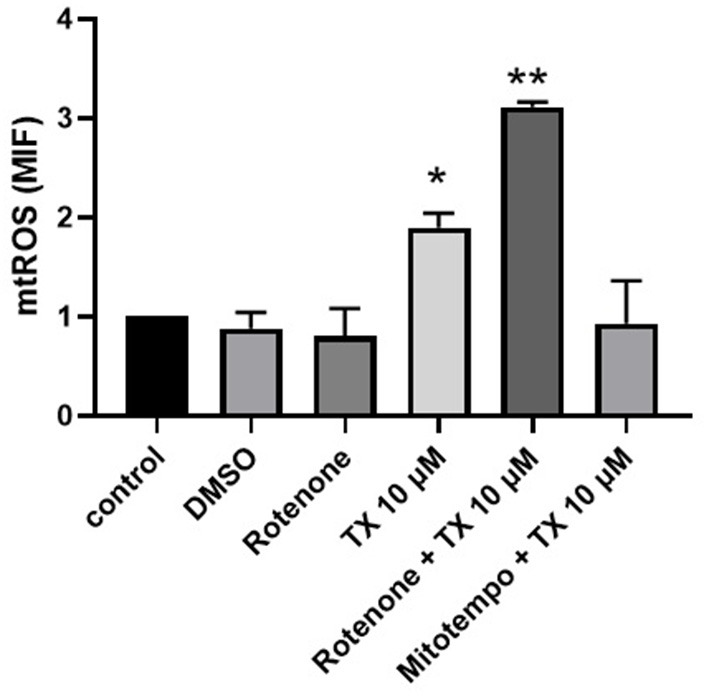
Tamoxifen induces mitochondrial ROS production. Equine neutrophils were stained with Mitosox and incubated with tamoxifen (10 uM), rotenone (10 uM), mitotempo(200 uM) or vehicle (DMSO 0.1%) for 30 min at 37°C. Where indicated, prior to the addition of tamoxifen (10 uM) the neutrophils were preincubated with rotenone (10 uM) or mitotempo (200 uM) for 30 min at 37°C. The mean fluorescence intensity (MIF) was analyzed by flow cytometry. The result corresponds to the average +/- S.E.M of 5 independent experiments.^*^p < 0.05; ^**^p < 0.01.

The effect of TX on mitochondrial ROS generation was assessed with the mitochondrial superoxide indicator mitosox. Cells treated with 10 uM TX showed a clear increase in mtROS levels, which was significantly enhanced when neutrophils were also pre-incubated with 10 uM rotenone (inhibitor of complex I of the electron chain). On the other hand, the effect of TX on mtROS was significantly reduced when cells were pre-incubated with the antioxidant mitoTEMPO (Figure 6).

## 4. Discussion

Our study results show that the stimulation of equine polymorphonuclear cells with TX triggers the production of NETs in healthy adult horses. In humans, TX has been shown to strongly promote NETs production in neutrophils by an estrogen receptor-independent pathway, which seems to be modulated by sphingolipid biosynthesis, which plays an important role in neutrophil activity, and is considered a ROS-independent Ceramide/PKCζ signaling pathway (27). Furthermore, the TX concentration of 10 μm was used in this study, based on the findings of Corriden et al. (27) in which he observed NETs in human neutrophils using this TX concentration.

During NET formation, the characteristic nuclear lobules of neutrophils disappear, and the chromatin expands while the cytoplasmic membrane remains intact. Two hours after stimulation, in this case with TX, this membrane ruptures, releasing chromatin decorated with antimicrobial proteins. The most marked morphological changes during netosis occur in the area, perimeter and shape of the nucleus. We observed that treatment with 10 μM TX induces spread-forming NETs (Figure 3). These *sprNETs* occupy three to five times the volume of condensed chromatin. Several proteins, as mentioned above, are attached to NETs, including histones and more than 30 components of primary and secondary granules, including components with bactericidal activity such as elastase, myeloperoxidase, cathepsin G, lactoferrin pentraxin 3, gelatinase, proteinase 3, LL37, peptidoglycan-binding proteins, and others with bactericidal activity capable of destroying virulence factors (4).

The importance of NETs in the progression of human asthma is controversial. Neutrophils do increase in sputum or BALF samples from severely asthmatic patients, either with acute exacerbation episodes or with persistent asthma (28). NETs can be deleterious to epithelial barrier function in the lung (29) and they can also promote proinflammatory mediator secretion from dendritic cells and protease release from neutrophils (30). Some authors suggest that NETs may act directly on airway epithelial cells, aggravating airway inflammation and respiratory clinical signs (31, 32). Proteases contained in NETs also activate proinflammatory cytokines, aggravating the inflammatory response even more (33). Together, all these studies suggest that NETs damage the airway epithelium, increase cytokine secretion, and worsen asthma. In asthmatic horses, NETs are found in BALF and correlate with clinical severity (34). Our previous studies mentioned above suggest that TX can modulate neutrophil functionality in equines; this would partly explain the beneficial effect of this drug in horses with airway inflammation. However, it was a surprise to observe that TX could trigger NETs formation. It seems that the inflammation-resolving effect of TX in horses with neutrophilic airway inflammation could be due to the other neutrophilic functional inhibitions described above, rather than through the inhibition of NETs triggering. It would certainly be necessary to perform an *in vivo* study of horses with severe asthma, treating them with TX and observing the behavior of neutrophils in BALF with respect to NETs formation.

On the other hand, another interesting finding in this study was that 5% of all NETs were *aggNETs*. In human gout, *aggNETs* produced by neutrophils induced by monosodium urate crystals (MSU) apparently orchestrate the resolution of inflammation through the degradation of inflammatory cytokines (35). The anti-inflammatory effect of *aggNETs* is produced by serine proteases which proteolytically degrade inflammatory cytokines and chemokines; this could have a potential impact on different inflammatory diseases associated with neutrophil accumulation (36). Moreover, Mahajan et al. (37) note that ocular rheuma, which forms after night-time sleep or after prolonged eye closure, favors a continuous infiltration of neutrophils on ocular surfaces. These authors demonstrated the coordinated sequence of events of neutrophil activity during eye closure, including infiltration, degranulation and NET formation, and found that *aggNETs* resolved neutrophilic inflammation by degrading cytokines and chemokines and thereby preventing overload, uncontrolled neutrophil recruitment and activation. As stated above, proteolytic degradation of inflammatory mediators by *aggNETs* results in the resolution of gouty arthritis in humans and mice (10). Therefore, the formation of *aggNETs* could be considered a type of negative feedback to promote the resolution of inflammation and as protection to contain neutrophilic inflammation, since independently of the stimulus that triggers *aggNETs* formation, at high enough cell density, proteolytic degradation of inflammatory mediators outpaces the release of inflammatory mediators, leading to resolution of inflammation as degradation is faster than release (36). However, some authors conclude that this mechanism (*aggNETs*) probably does not have a major role *in vivo* in the context of MSU-crystal-induced footpad or joint inflammation in mice (38).

Equine neutrophils reactive oxygen species (ROS) production was dampened after treatment with TX (16). Lim et al. (39) had previously reported an inhibitory effect of TX 5 μM on H_2_O_2_ production in TPA-stimulated neutrophils from healthy humans. Other authors also observed a profound inhibition of ROS production in stimulated human neutrophils after treatment with TX (27, 40). Thus, the source of ROS that triggers NETs by TX should come from the mitochondria. Likewise, Albornoz et al. (15) showed that tamoxifen, N-desmethyltamoxifen and endoxifen depolarize the mitochondrial membrane and activate caspase-3 in healthy equine neutrophils *in vitro*. These results suggest that TX has the capacity to produce alterations in mitochondria, probably by stimulating the release of mtROS. Mitochondria have important roles in cellular function, including participation in energy generation and modulation of apoptosis (41). In addition, mitochondria produce large amounts of ROS and are important participants in redox-dependent intracellular signaling (42). For a long time, it was believed that mitochondria do not play a significant role in the functioning of neutrophils, since their content in these cells is low, energy supply is supported by glycolysis, and NADPH oxidase is the main source of ROS. However, mitochondrial dysfunction and oxidative stress in and around mitochondria have been implicated in pathogenic mechanisms, including inflammation and autoimmune reactions (43). Mitochondria are involved in the transmission of signals that determine main neutrophil responses to pathogens (44). It was found that mitochondrial ROS are involved in the activation of NADPH oxidase and in the induction of NETosis caused by various stimuli (45, 46). Therefore, the present study wanted to test whether TX can trigger mtROS, which would partly explain the formation of NETs. For this, rotenone was used as a strong inhibitor of complex I of the mitochondrial respiratory chain. The mechanism of action comprises inhibition of electron transfer from the iron-sulfur centers in complex I to ubiquinone, leading to a blockade of oxidative phosphorylation with limited synthesis of ATP (47). Furthermore, incomplete electron transfer to oxygen could lead to ROS formation. This rotenone-induced ROS production, with an assumed damage of mitochondria components, including mitochondrial DNA, can eventually lead to apoptosis (48). However, at our incubation times rotenone did not influence significative mtROS release; but in the presence of tamoxifen there was a synergistic effect between the two drugs. Activation of NADPH oxidase by mtROS was first demonstrated by Dikalov and coworkers (28) in endothelial cells by mitoTEMPO. This drug is a mitochondria-targeted antioxidant that prevents mitochondrial oxidative damage. Our results indicate that the use of mitoTEMPO reduces the levels of mtROS produced by TX in equine neutrophils (Figure 5). This latter result may suggest that mitochondria play an important role in the formation of NETs in the presence of TX. However, a weakness of this study was the non-performance of immunofluorescence to observe the effect of TX on NETs production in the presence of rotenone and mitoTEMPO.

## 5. Conclusion

In conclusion, neutrophils and NETs play an important role in the defense against invading pathogens, possibly in the resolution of inflammation. NETs and NET-associated proteases trap and clear pathogens; *aggNETs* could have anti-inflammatory effects by clearing and degrading inflammatory cytokines. TX triggered NETs formation (mainly *sprNEts* and to a very low percentage *aggNETs*). Furthermore, this study suggests that TX- induced NETs in equine neutrophils may be triggered by mtROS. However, disruption of the delicate balance between the formation, degradation and elimination of NETs may contribute to the development and pathogenesis of various inflammatory diseases such as equine asthma. NETs and their components interact with other cells of the innate and adaptive immune system and thus modulate immune responses. Therefore, it is imperative to perform *in vivo* studies to see the real effect of TX on NET production.

## Data availability statement

The raw data supporting the conclusions of this article will be made available by the authors, without undue reservation.

## Ethics statement

All procedures were approved by the Universidad Austral de Chile Bioethics Committee for the Use of Animals in Biomedical Research (approval resolution n° 348/2019).

## Author contributions

CS, KB, JS, and AA performed the experiments. JQ, PA, and RB performed the data analysis. RB, CH, PE, BU, and GM conceived and designed the experiments. GM and BU wrote the manuscript. All authors contributed to the article and approved the submitted version.
